# Supplementary material for: Machine Learning Accelerates Crystallization for Structure Determination
Source: Angew Chem Int Ed Engl. 2026 May 4;65(25):e1218503. doi: 10.1002/anie.1218503 (PMC13266958; doi:10.1002/anie.1218503)

checkCIF/PLATON report

Structure factors have been supplied for datablock(s) lcz055

THIS REPORT IS FOR GUIDANCE ONLY. IF USED AS PART OF A REVIEW PROCEDURE FOR PUBLICATION, IT SHOULD NOT REPLACE THE EXPERTISE OF AN EXPERIENCED CRYSTALLOGRAPHIC REFEREE.

No syntax errors found.   CIF dictionary   Interpreting this report

Datablock: lcz055

Bond precision: C-C = 0.0125 A   Wavelength=1.54184

Cell:	a=12.4662(3)	b=13.1607(2)	c=20.7819(2)
	alpha=92.289(1)	beta=95.027(1)	gamma=91.149(1)
Temperature:	100 K		

	Calculated	Reported
Volume	3392.79(10)	3392.78(10)
Space group	P -1	P -1
Hall group	-P 1	-P 1
Moiety formula	C37 H23 Ag3 F18 N6 O6, C15 H3 Ag3 F18 N6	2(C15 H3 Ag3 F18 N6), 2(C11 H10 O3)
Sum formula	C52 H26 Ag6 F36 N12 O6	C52 H26 Ag6 F36 N12 O6
Mr	2246.07	2246.07
Dx,g cm-3	2.199	2.199
Z	2	2
Mu (mm-1)	15.074	15.074
F000	2152.0	2152.0
F000’	2162.49	
h,k,lmax	15,16,26	15,16,26
Nref	13981	13599
Tmin,Tmax	0.105,0.104	0.727,1.000
Tmin’	0.022	

Correction method= # Reported T Limits: Tmin=0.727 Tmax=1.000
AbsCorr = MULTI-SCAN

Data completeness= 0.973 Theta(max)= 74.999

R(reflections)= 0.0599(10326) wR2(reflections)= 0.1409(13599)

S = 1.056 Npar= 1007

The following ALERTS were generated. Each ALERT has the format
test-name_ALERT_alert-type_alert-level.
Click on the hyperlinks for more details of the test.

Alert level C

```
PLAT042_ALERT_1_C Calc. and Reported MoietyFormula Strings  Differ     Please Check
             Calc: C37 H23 Ag3 F18 N6 O6, C15 H3 Ag3 F18 N6
             Rep.: 2(C15 H3 Ag3 F18 N6), 2(C11 H10 O3)
PLAT213_ALERT_2_C Atom F00L            has ADP max/min Ratio .....        3.3 prolat
PLAT213_ALERT_2_C Atom F01I            has ADP max/min Ratio .....        3.5 prolat
PLAT234_ALERT_4_C Large Hirshfeld Difference F027     --C033     .       0.17 Ang.
PLAT250_ALERT_2_C Large U3/U1 Ratio for <U(i,j)> Tensor(Resd    1)        2.2 Note
PLAT342_ALERT_3_C Low Bond Precision on  C-C Bonds ..............     0.0125 Ang.
PLAT906_ALERT_3_C Large K Value in the Analysis of Variance ......     15.856 Check
PLAT906_ALERT_3_C Large K Value in the Analysis of Variance ......      3.545 Check
PLAT911_ALERT_3_C Missing FCF Refl Between Thmin & STh/L=    0.600         94 Report
              12  7  7,    7  9  7,   12  5  8,   12  6  8,    7  7  8,    5 13  8,
              11  3  9,    6 12  9,    5 13  9,   11  4 10,   -1 12 10,    3-12 11,
               5 10 11,   -1 12 11,    1-12 12,  -13  3 13,    7  6 13,   -1 12 13,
               7  6 14,  -11  6 15,  -10  7 15,   -8  8 15,   -7 10 15,   -8  9 16,
             -10 -3 18,   -4 10 18,   -1 -6 19,   -1  5 19,    0  7 19,   -4  9 19,
                  (   64 More Missing: see the .ckf listing file)
PLAT934_ALERT_3_C Number of (Iobs-Icalc)/Sigma(W) > 10 Outliers ..          1 Check
              -2 -7 22,
PLAT971_ALERT_2_C Check Calcd Resid. Dens.  1.05Ang From Ag04            1.62 eA-3
```

Alert level G

```
PLAT003_ALERT_2_G Number of Uiso or U(i,j)  Restrained non-H-Atoms         17 Report
PLAT083_ALERT_2_G SHELXL Second Parameter in WGHT  Unusually Large      38.14 Why ?
PLAT154_ALERT_1_G The s.u.'s on the Cell Angles are Equal ..(Note)      0.001 Degree
PLAT171_ALERT_4_G The CIF-Embedded .res File Contains EADP Records          1 Report
PLAT186_ALERT_4_G The CIF-Embedded .res File Contains ISOR Records          4 Report
PLAT242_ALERT_2_G Low    'MainMol' Ueq as Compared to Neighbors of       C022 Check
PLAT242_ALERT_2_G Low    'MainMol' Ueq as Compared to Neighbors of       C023 Check
PLAT242_ALERT_2_G Low    'MainMol' Ueq as Compared to Neighbors of       C026 Check
PLAT242_ALERT_2_G Low    'MainMol' Ueq as Compared to Neighbors of       C02A Check
PLAT242_ALERT_2_G Low    'MainMol' Ueq as Compared to Neighbors of       C02H Check
PLAT242_ALERT_2_G Low    'MainMol' Ueq as Compared to Neighbors of       C02X Check
PLAT242_ALERT_2_G Low    'MainMol' Ueq as Compared to Neighbors of       C02O Check
PLAT242_ALERT_2_G Low    'MainMol' Ueq as Compared to Neighbors of       C02V Check
PLAT242_ALERT_2_G Low    'MainMol' Ueq as Compared to Neighbors of       C030 Check
PLAT242_ALERT_2_G Low    'MainMol' Ueq as Compared to Neighbors of       C031 Check
PLAT242_ALERT_2_G Low    'MainMol' Ueq as Compared to Neighbors of       C033 Check
PLAT242_ALERT_2_G Low    'MainMol' Ueq as Compared to Neighbors of       C034 Check
PLAT434_ALERT_2_G Short Inter HL..HL Contact F009     ..F028     .       2.79 Ang.
                                                  x,y,z  =      1_555 Check
```

```
PLAT434_ALERT_2_G Short Inter HL..HL Contact F00F     ..F00V     .       2.80 Ang.
                                                       x,y,z  =     1_555 Check
PLAT720_ALERT_4_G Number of Unusual/Non-Standard Labels .........        138 Note
              Ag01    Ag02    Ag03    Ag04    Ag05    Ag06    F007    F008
              F009    F00A    F00B    O00C    F00D    F00E    F00F    O00G
              O00H    F00I    F00J    F00K    F00L    O00M    F00N    F00O
              O00P    N00Q    F00R    O00S    N00T    N00U    F00V    F00W
              F00X    N00Y    N00Z    F010    F011    N012    F013    F014
              N015    N016    N017    N018    F019    N01A    C01B    N01C
              F01D    F01E    F01F    F01G    C01H    F01I    C01J    H01J
              C01K    C01L    F01M    F01N    F01O    C01P    C01Q    C01R
              H01R    C01S    H01S    C01T    C01U    C01V    C01W    H01W
              C01X    H01X    C01Y    H01Y    C01Z    H01Z    C020    F021
              C022    C023    C024    C025    C026    F027    F028    C029
              C02A    C02B    C02C    H02C    C02D    F02E    C02F    C02G
              H02G    C02H    C02I    H02A    H02B    H02D    C02J    H02E
              H02F    H02H    C02K    H02I    H02J    H02K    C02L    H02L
              C02M    H02M    C02N    H02N    C02O    C02P    C02Q    C02R
              H02R    C02S    H02S    C02T    F02U    C02V    C02W    C02X
              C02Y    C02Z    C030    C031    C032    H03A    H03B    H03C
              C033    C034
PLAT860_ALERT_3_G Number of Least-Squares Restraints ............        102 Note
PLAT883_ALERT_1_G Absent Datum for _atom_sites_solution_primary ..     Please Do !
PLAT910_ALERT_3_G Missing FCF Reflection(s) Below Theta(Min)[Deg]=      3.36 Note
                0  0  1,
PLAT912_ALERT_4_G Missing # of FCF Reflections Above STh/L=  0.600        287 Note
PLAT933_ALERT_2_G Number of HKL-OMIT Records in Embedded .res File         85 Note
              -10  8 16,  -9  9 15,  -8  9 16,  -8 10 16,  -7 10 15,  -7 10 16,
               -6  8 20,  -6  9 19,  -5  0 21,  -5  7 22,  -5  8 21,  -5  9 19,
               -5  9 20,  -5 10 18,  -4 -5 21,  -4  2 22,  -4  3 20,  -4  7 22,
               -4  8 21,  -4  9 19,  -4 10 18,  -3 -6 21,  -3 -6 22,  -3  0 24,
               -3  1 23,  -3  1 24,  -3  2 21,  -3  2 22,  -3  2 23,  -3  3 20,
               -3  3 21,  -3  3 22,  -3  4 20,  -2 -1 25,  -2  0 24,  -2  0 25,
               -2  1 23,  -2  1 24,  -2  2 22,  -2  2 23,  -2  2 24,  -2  3 20,
               -2  3 21,  -2  3 22,  -2  3 23,  -2  4 20,  -2  6 22,  -2  7 21,
               -1 -1 25,  -1 -1 26,
PLAT941_ALERT_3_G Average HKL Measurement Multiplicity ..........        2.7 Low
PLAT967_ALERT_5_G Note: Two-Theta Cutoff Value in Embedded .res ..      150.0 Degree
PLAT969_ALERT_5_G The 'Henn et al.' R-Factor-gap value ..........      2.553 Note
              Predicted wR2: Based on SigI**2  5.52 or SHELX Weight 13.33
PLAT978_ALERT_2_G Number C-C Bonds with Positive Residual Density.          0 Info
```

```
   0 ALERT level A = Most likely a serious problem - resolve or explain
   0 ALERT level B = A potentially serious problem, consider carefully
  11 ALERT level C = Check. Ensure it is not caused by an omission or oversight
  29 ALERT level G = General information/check it is not something unexpected

   3 ALERT type 1 CIF construction/syntax error, inconsistent or missing data
  22 ALERT type 2 Indicator that the structure model may be wrong or deficient
   8 ALERT type 3 Indicator that the structure quality may be low
   5 ALERT type 4 Improvement, methodology, query or suggestion
   2 ALERT type 5 Informative message, check
```

Validation response form

Please find below a validation response form (VRF) that can be filled in and pasted into your CIF.

```
# start Validation Reply Form
_vrf_PLAT042_lcz055
;
PROBLEM: Calc. and Reported MoietyFormula Strings  Differ     Please Check
RESPONSE: ...
;
_vrf_PLAT213_lcz055
;
PROBLEM: Atom F00L            has ADP max/min Ratio .....        3.3 prolat
RESPONSE: ...
;
_vrf_PLAT234_lcz055
;
PROBLEM: Large Hirshfeld Difference F027     --C033     .       0.17 Ang.
RESPONSE: ...
;
_vrf_PLAT250_lcz055
;
PROBLEM: Large U3/U1 Ratio for <U(i,j)> Tensor(Resd    1)        2.2 Note
RESPONSE: ...
;
_vrf_PLAT342_lcz055
;
PROBLEM: Low Bond Precision on  C-C Bonds ..............     0.0125 Ang.
RESPONSE: ...
;
_vrf_PLAT906_lcz055
;
PROBLEM: Large K Value in the Analysis of Variance ......     15.856 Check
RESPONSE: ...
;
_vrf_PLAT911_lcz055
;
PROBLEM: Missing FCF Refl Between Thmin & STh/L=    0.600         94 Report
RESPONSE: ...
;
_vrf_PLAT934_lcz055
;
PROBLEM: Number of (Iobs-Icalc)/Sigma(W) > 10 Outliers ..          1 Check
RESPONSE: ...
;
_vrf_PLAT971_lcz055
;
PROBLEM: Check Calcd Resid. Dens.  1.05Ang From Ag04           1.62 eA-3
RESPONSE: ...
;
# end Validation Reply Form
```

It is advisable to attempt to resolve as many as possible of the alerts in all categories. Often the minor alerts point to easily fixed oversights, errors and omissions in your CIF or refinement strategy, so attention to these fine details can be worthwhile. It is up to the individual to critically assess their own results and, if necessary, seek expert advice.

PLATON version of 17/09/2025; check.def file version of 17/09/2025

duplicate check

No duplication found

Datablock lcz055 - ellipsoid plot

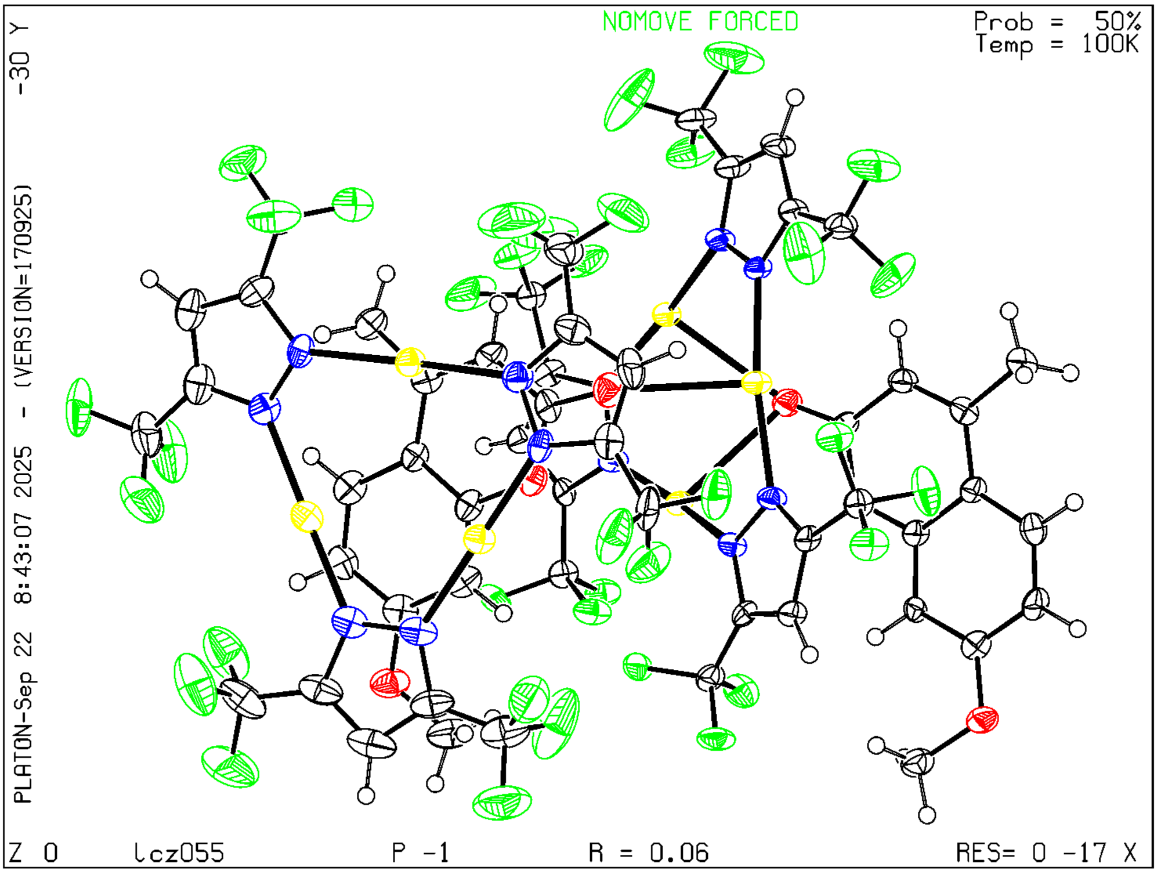

Supplement: Supplementary file 2 — Supporting File 2: anie72462‐sup‐0002‐DataFile.zip. [file ANIE-65-e1218503-s002.zip › supplementary_materials_crystallographic_data submit.zip!/cif_and_checkcif(1-114)/Ag3Pz3_55/Ag3Pz3_55_checkcif.pdf]
